# Correlation of Computed Tomography (CT) Severity Score With Laboratory and Clinical Parameters and Outcomes in Coronavirus Disease 2019 (COVID-19)

**DOI:** 10.7759/cureus.52324

**Published:** 2024-01-15

**Authors:** Bharath K Pamulapati, Ramesh K Nanjundappa, Bala S Chandrabhatla, Sumayya U Roohi, Sushrut Palepu

**Affiliations:** 1 Critical Care Medicine, Citizens Specialty Hospital, Hyderabad, IND; 2 Critical Care Medicine, Continental Hospitals, Hyderabad, IND

**Keywords:** neutrophil-to-lymphocyte ratio (nlr), serum ferritin, covid-19 outcomes, prognostication, length of stay, il-6, d-dimer, ct severity score, covid-19

## Abstract

Background: Coronavirus disease 2019 (COVID-19) is a potentially lethal respiratory illness caused by a newly identified coronavirus called severe acute respiratory syndrome coronavirus 2 (SARS-CoV-2). Given the novelty of the virus, high caseloads, and increasing turnaround time for reverse transcriptase-polymerase chain reaction (RT-PCR) results, accurate information about the clinical course and prognosis of individual patients was largely unknown. This has forced physicians all over the world to brainstorm attempts to come up with reliable indicators like chest high-resolution computed tomography (HRCT) for any changes suggestive of COVID-19; surrogate laboratory parameters such as C-reactive protein (CRP), ferritin, D-dimer, lactate dehydrogenase (LDH), or interleukin-6 (IL-6) for assessing the severity of the disease; and other organ-specific tests to identify the multiorgan involvement in severe-to-critical COVID-19. Chest computed tomography (CT) scans play a significant role in the management of COVID-19 disease and serve as an indicator of disease severity and its possible outcome, which might help in the early identification of patients who might need critical care and earlier prognostication.

Methods: A retrospective observational study was conducted at a single center in a level 3 critical care unit (CCU) of a 750-bed teaching hospital in Hyderabad, Telangana, India, over a period of six months. All RT-PCR-positive COVID-19 patients admitted to the CCU with CT chest performed within 24 hours of admission were screened for eligibility for this study. CT severity scoring was based on chest HRCT or CT.

Results: Of the 110 patients, a majority (36.36%) were aged between 61 and 70 years. The mean age of our study population was 59.65±11.88 years. Of the 110 patients, the majority were admitted to the hospital for 22-28 days (24.55%), followed by 8-14 days (22.72%), and 21.82% were admitted for one day. Of the 110 patients, a majority were admitted to the CCU for seven days (41.82%), followed by 15-21 days (24.55%); and 19.09% were admitted for 8-14 days. Most of the patients were discharged (65.45%), and we had a 34.55% mortality rate in our study. We found a significant association between chest CT severity score (CTSS) and the age of the patient, duration of hospital stay, and duration of CCU stay using multivariate regression analysis.

Conclusion: CTSS could be greatly helpful for the screening and early identification of the disease, especially in those patients awaiting an RT-PCR report or with negative RT-PCR, which would lead to appropriate isolation and treatment measures. Early detection could also help assess the progression of the disease, alter the course of management at the earliest point possible, and improve the prognostication of COVID-19 patients.

## Introduction

Coronavirus disease 2019 (COVID-19) infection has become a global health threat, causing an ongoing pandemic with significant impact on social, political, and healthcare systems. The huge number of cases has led to many emergencies and overwhelmed inpatient care. With sustained transmission to all six continents, the World Health Organization (WHO) declared COVID-19 a global pandemic in March 2020 [1-3]. COVID-19 is a potentially lethal respiratory illness caused by a newly identified coronavirus called severe acute respiratory syndrome coronavirus 2 (SARS-CoV-2) [4]. The clinical presentations vary from asymptomatic carriers to patients who need invasive ventilatory support. The need for critical care unit (CCU) admissions and high mortality rates made it a global challenge. The reverse transcriptase-polymerase chain reaction (RT-PCR) test of nasopharyngeal or throat swab has been the diagnostic test used as the standard for disease confirmation [5]. RT-PCR is a powerful tool in diagnosing the disease; however, a small but significant proportion of false-negative results has been reported. False-negative cases have important implications for the isolation and risk of transmission by infected people and for the management of COVID-19 [6].

Given the novelty of the virus, high caseloads, and increasing turnaround time for RT-PCR results, accurate information about the clinical course and prognosis of individual patients is still largely unknown. This lack of knowledge has forced physicians all over the world to brainstorm to develop reliable indicators like chest high-resolution computed tomography (HRCT) for any changes suggestive of COVID-19; surrogate laboratory parameters like C-reactive protein (CRP), ferritin, D-dimer, lactate dehydrogenase (LDH), or interleukin-6 (IL-6) for assessing the severity of the disease; and other organ-specific tests to identify the multiorgan involvement in severe-to-critical COVID-19. Patients with mild COVID-19 infection are hospitalized in the usual isolation ward, of which a small subset develop severe disease. Approximately 5% of infected patients are critical cases who require admission to the CCU. In these patients, COVID-19 can be complicated by acute respiratory distress syndrome, septic shock, and multiorgan failure, including kidney and heart failure [7]. D-dimer is a biomarker for thrombotic disorders and has been used as a potential prognostic indicator in COVID-19 patients [8]. Ferritin is an acute-phase reactant protein, and its serum concentrations can be elevated, irrespective of a change in iron stores, by infection or inflammation [9]. A valuable marker to predict the possibility of aggravation of mild forms of COVID-19 to severe forms could be elevated CRP [10]. Non-contrast HRCT chest imaging plays a pivotal role in early disease detection, particularly in patients with false-negative RT-PCR results, as well as in managing and monitoring the course of the disease [11].

Chest CT scans play a significant role in the management of COVID-19. They are an indicator of disease severity and a possible outcome predictor. The CT severity score (CTSS) is directly correlated with inflammatory markers, average length of hospital stay, and oxygen requirements in COVID-19-positive patients [5]. Clinicians and radiologists should familiarize themselves with CT findings in COVID-19 patients for diagnosing and estimating the prognosis of COVID-19 disease. An early suspicion of COVID-19 pneumonia can be generated with the help of chest CT images in the correct clinical setting. Bilateral ground-glass opacities (GGOs) or consolidation in chest CT images should cue radiologists to suggest a possible COVID-19 diagnosis [11]. Authors of numerous international studies have attempted to correlate the clinical profiles, laboratory parameters, and chest HRCT in their respective patient population with the course of the disease. We conducted this study to evaluate our patients based on various clinical, radiological, and laboratory parameters to correlate outcomes with CTSS at presentation, which might help in the early identification of patients who might need intensive care, and facilitate earlier prognostication.

## Materials and methods

A retrospective observational study was conducted at Continental Hospitals in Hyderabad, Telangana, India, over a period of six months. The study subjects were observed, and the outcomes were measured without attempting to make changes to affect the outcome. Inferences were drawn from the sample of population under study. All the study subjects who fulfilled the inclusion criteria as described in Table 1 were included in the study, achieving a sample of 110.

**Table 1:** Inclusion and exclusion criteria COVID-19: coronavirus disease 2019; RT-PCR: reverse transcriptase-polymerase chain reaction; CCU: critical care unit; ER/IPD: emergency room/inpatient department; CT: computed tomography

Inclusion criteria	Exclusion criteria
Age more than 18 years	Age less than 18 years
Diagnosed with COVID-19 through a positive RT-PCR test	RT-PCR test was negative
Shifted to CCU from ER/IPD in view of increased disease severity with CT scan done around 24 hours of shifting to CCU	
Only those patients whose admission CT is performed at our institute	Underwent chest CT outside the hospital and shifted to our center

The study proforma contained the following details: Part 1: patient admission details; Part 2: medical history; Part 3: laboratory parameters; and Part 4: chest CT imaging and scores. CT severity scoring was based on chest HRCT or CT pulmonary angiogram on admission by a qualified radiologist in the department of radiology at our institute. Chest CT or CT pulmonary angiography quantifies the extent of lung involvement using a 25-point scoring system that was studied in patients with COVID-19 by Yang et al. [12]. This system was adapted to COVID-19 by an expert consensus panel from the Radiological Society of North America as explained in Table 2 and Table 3. Patient data such as demographics, clinical course, laboratory data, and outcome used in the study were drawn from the electronic medical record (EMR). Laboratory parameters were collected at admission and 24 hours after the initiation of treatment, and one value was taken for every week of CCU stay, the most abnormal value of the week. Correlation of CTSS with various laboratory parameters, clinical events, and non-mortality outcome measures was done.

**Table 2:** CT severity scoring CT: computed tomography

Score	Definition
0	None
1	<5% of lobe
2	5-25% of lobe
3	26-49% of lobe
4	50-75% of lobe
5	>75% of lobe

**Table 3:** Sum of scores Sum of scores of individual lobes gives the overall severity

Total score (numerical)	Severity (category)
7 or less	Mild
8-17	Moderate
18 or more	Severe

## Results

Patients admitted to the level 3 CCU with a diagnosis of COVID-19 at Continental Hospitals, Hyderabad, who satisfied the inclusion criteria during the study period were included in the study. A total of 110 COVID-19 patients were enrolled as the study population, and statistical analysis revealed the following observations.

**Figure 1:**
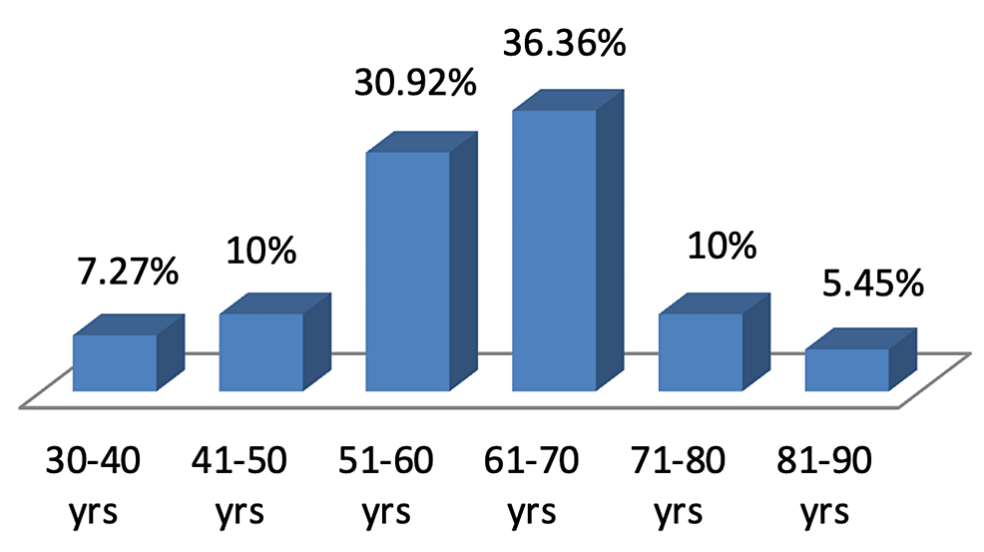
Demographics of the study Data is expressed as frequency of distribution in percentages

**Figure 2:**
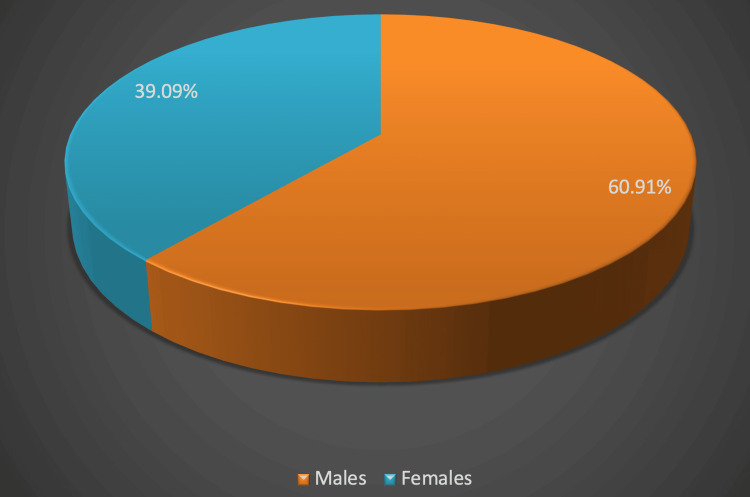
Sex distribution among the study population Sex distribution is expressed as percentages

**Figure 3:**
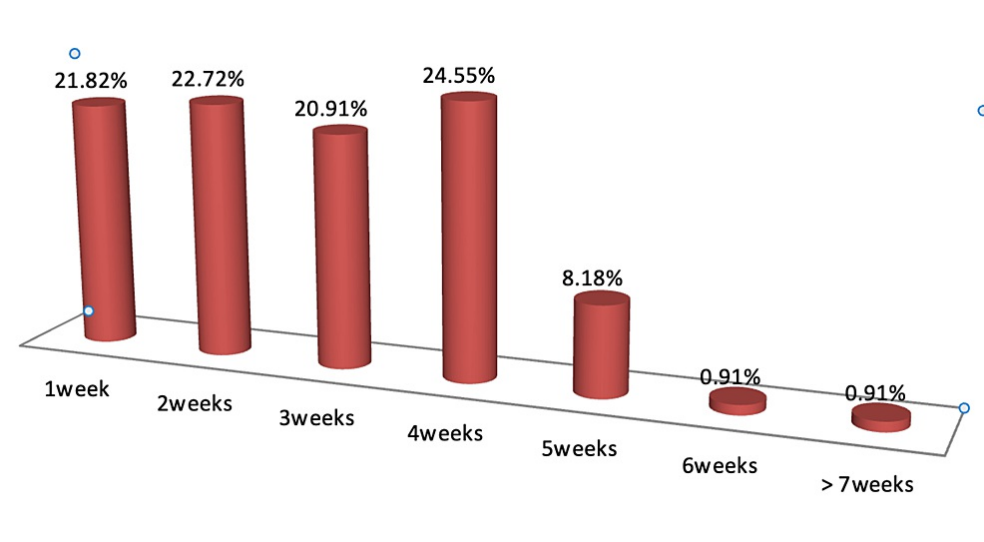
Length of hospital stay Frequency distribution of length of hospital stay of patients is expressed as percentages

**Figure 4:**
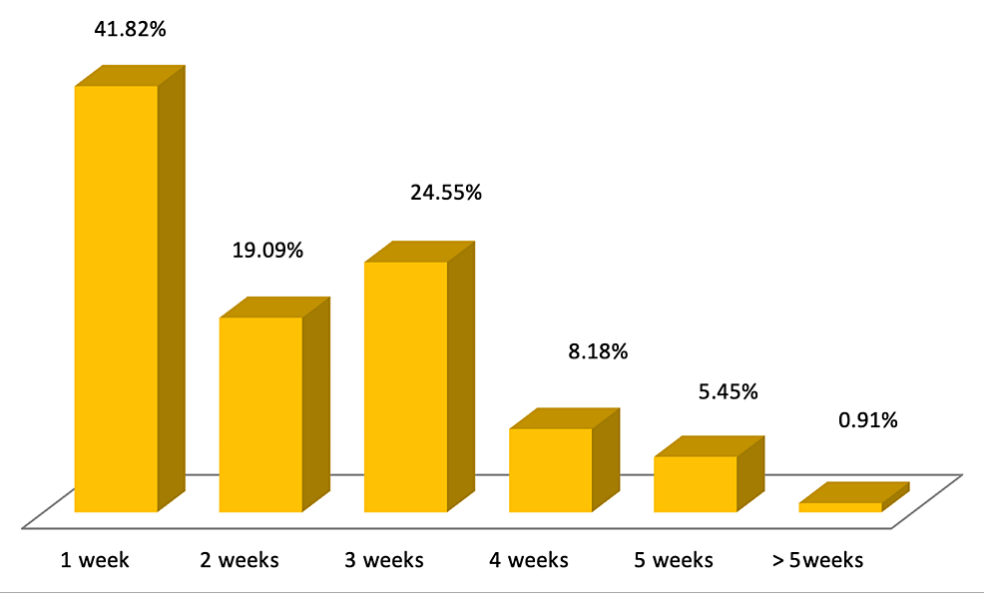
Length of CCU stay Frequency distribution of length of CCU stay is expressed as percentages CCU: critical care unit

**Figure 5:**
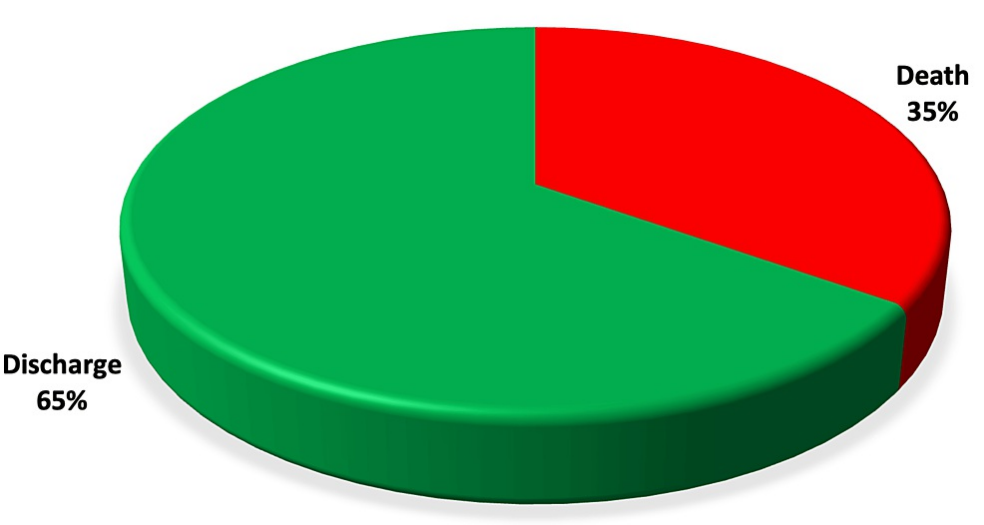
Outcomes among the study population Outcomes are expressed as percentages

Figure 1 explaining about the demographics of the study revealed that the mean age of the study population was 59.65±11.88 years with a majority (36.36%) being aged between 61 and 70 years. Of the 110 patients studied, 67 (60.91%) were males, and 43 (39.09%) were females as shown in Figure 2. The length of hospital stay in the study population revealed that majority of the study population were admitted in the hospital for 22-28 days (24.55%), followed by 8-14 days (22.72%), and 21.82% were admitted for only one day as shown in Figure 3. Among the patients admitted to the CCU, the majority were in CCU for seven days (41.82%), followed by 15-21 days (24.55%), and 19.09% were admitted for 8-14 days as shown in Figure 4. Of the overall distribution of different comorbidities in our study, the majority of patients had diabetes (n=44, 40%), followed by hypertension (n=14, 12.73%) as explained in Table 4. The majority of the patients were discharged (65.45%), and we had a 34.55% mortality rate in our study. The mortality rate observed among the study population is not a reflection of actual COVID-19 mortality at the institute as depicted in Figure 5.

**Table 4:** Distribution of comorbidities Includes multiple answers like the same patient can have both diabetes and hypertension

Comorbidities	Frequency	Percentage
Hypertension	14	12.73%
Diabetes	44	40%
Coronary artery disease	8	7.27%
Hypothyroidism	2	1.82%
Others	4	3.64%

Table 5 presents the correlation between CTSS and various laboratory parameters and Acute Physiology and Chronic Health Evaluation (APACHE) scores in patients with COVID-19. There were statistically significant correlations between CTSS, neutrophil-to-lymphocyte ratio (NLR), CRP, D-dimer, and LDH levels among COVID-19 patients. Ferritin, IL-6, and APACHE II scoring did not show statistical correlation with CTSS. Multivariate regression analysis shows a significant association between CTSS and the laboratory parameters NLR and LDH as explained in Table 6. As explained in Table 7, 11 patients with a CTSS of less than 8, 25 patients with a CTSS of between 8 and 14, and 74 patients with a CTSS of more than 15 were admitted to the CCU. There is no statistically significant association between CTSS and outcome (discharge or death). As the CTSS increased, the length of hospital and length of CCU stay also increased, as did the need for invasive ventilation. All of these were statistically significant. We found a significant association between CTSS and the age of the patient, duration of hospital stay, and duration of ICU stay.

**Table 5:** Correlation of CTSS with laboratory parameters and APACHE II score p<0.05 is considered statistically significant CTSS: computed tomography severity score; NLR: neutrophil-to-lymphocyte ratio; CRP: C-reactive protein; LDH: lactate dehydrogenase; IL-6: interleukin-6; APACHE: Acute Physiology and Chronic Health Evaluation

Correlation	Statistics	NLR	CRP	Ferritin	D-dimer	LDH	IL-6	APACHE II
CTSS	Correlation coefficient	0.318	0.220	0.111	0.236	0.510	-0.008	0.033
	p-value	0.001	0.026	0.260	0.020	<0.001	0.951	0.734

**Table 6:** Multivariate regression analysis of laboratory parameters in COVID-19 with CTSS p<0.05 is considered statistically significant OR: odds ratio; CI: confidence interval; NLR: neutrophil-to-lymphocyte ratio; CRP: C-reactive protein; LDH: lactate dehydrogenase; COVID-19: coronavirus disease 2019; CTSS: computed tomography severity score

Parameter	OR	95% CI	p-value
NLR	0.131	0.014, 0.249	0.029
CRP	0.007	-0.004, 0.018	0.200
D-dimer	-0.003	-0.012, 0.005	0.427
LDH	0.012	0.007, 0.016	0.000

**Table 7:** Multivariate regression analysis of age and length of hospital and ICU stay with CTSS in COVID-19 p<0.05 is considered statistically significant OR: odds ratio; CI: confidence interval; ICU: intensive care unit; CTSS: computed tomography severity score; COVID-19: coronavirus disease 2019

Parameter	OR	95% CI	p-value
Age	-0.205	-0.288, -0.122	0.000
Duration of hospital stay	-0.307	-0.566, -0.048	0.021
Duration of ICU stay	0.388	0.106, 0.669	0.008

## Discussion

The present study attempts to provide an outline distribution of age, gender, and comorbidity in patients with COVID-19. Our goal was to study the correlation between clinical parameters, laboratory parameters, and outcomes in COVID-19 patients with CTSS. Chest imaging was recommended by the WHO as part of a diagnostic workup in COVID-19 patients in situations in which RT-PCR testing is either not available or too delayed or when there is a strong clinical suspicion of COVID-19 even when RT-PCR testing is negative [13]. Chest CT is a widely used tool for early screening and diagnosing patients with suspected COVID-19. The common chest CT characteristics exhibited by COVID-19 patients as mentioned in some previous studies include GGOs and consolidation. Results from such studies also showed that most apparent abnormalities on chest CT were still observable for 10 days but disappeared at 14 days after the initial onset of symptoms [14,15]. However, reports regarding the long-term longitudinal development of imaging features and correlation with pathological assessment in COVID-19 patients are still lacking. Normal chest radiographs early in the course of disease can lead to many false-negative interpretations [16]. A timely and accurate diagnosis is important as the first step in disease containment and patient management. HRCT is more sensitive than chest radiography in identifying early parenchymal changes. Therefore, chest CT has become a lead diagnostic test of choice during the COVID-19 pandemic [17,18]. A study by Zhou et al. reported that individual scores in each lung and the total CTSS were higher in severe COVID-19 when compared with mild cases (p=-0.05). The optimal CTSS threshold for identifying severe COVID-19 was 19.5 (area under curve=0.892), with 83.3% sensitivity and 94% specificity [19].

COVID-19 patients with critical disease present CT features that reflect a more advanced and severe presentation and involve a greater reduction in respiratory exchanges. The combined presence of consolidation and GGO is a sign of alveolar damage at different stages, whereas interlobular thickening is a sign of interstitial pathology. In the earlier and less severe phases of the disease, an incomplete alveolar filling is usually seen and is depicted as GGO on CT [20]. It has been suggested that the enlargement of subsegmental vessels could reflect the hyperaemia induced by SARS-CoV-2 infection and might be the result of pro-inflammatory factors' release [21]. However, there remains no clear evidence of the actual role of this imaging feature nor of the reason it is more frequent in critically ill patients. 

Age and gender

In our study, 36.36% of the study population was aged between 61 and 70 years. A statistical significance was noted between higher CTSS of 15-25 in the elder population compared with the younger age group (p<0.01). Similar observations were reported by Liu et al. among COVID-19 patients aged >60 years with a higher rate of respiratory failure, requiring more prolonged treatment than those aged <60 years. This further demonstrates that elderly COVID-19 patients had much more severe disease than the younger group, as supported by an increased level of inflammatory markers [22]. A study by Dangis et al. showed a significant correlation (p<0.05) of CTSS in males compared with females, which could be attributed to a possible protective effect of estrogen among females [23]. However, although our study had a majority of male subjects (61.3%), we did not assess its correlation with gender distribution. Bhandari et al. reported that most COVID-19 patients in their study group were in their fourth to sixth decades of life with a mean age of 50.40 years and that males were affected more than females. Our study group had an average sex ratio of 0.69 [24].

Comorbidities

In our study, the distribution of comorbidities such as diabetes, hypertension, and cardiovascular disease among the study subjects was 40%, 12.7%, and 7.2%, respectively. Similar results with diabetes, hypertension, and cardiovascular disease were reported by Wang et al. [10]. Richardson et al. found that the most common comorbidities among admitted COVID-19 patients were hypertension (56.6%), obesity (41.7%), and diabetes (33.8%). However, among those who died, patients with diabetes were more likely to have received invasive mechanical ventilation than those who did not have diabetes. Those with hypertension were less likely to have received invasive mechanical ventilation or care in the CCU than those without hypertension [25]. Many existing studies suggest that the presence of risk factors, particularly hypertension, diabetes, and lung and coronary artery diseases, indicates a poor prognosis [24,26]. Although a study by Saeed et al. did not find a statically significant correlation between the presence of risk factors and CTSS, there was, however, a significant correlation (p<0.0001) between CCU admission and the presence of risk factors [1].

Laboratory findings

CRP can be used as a predictive marker of disease progression and to identify disease progression by following trends, as suggested by several studies. Significant correlations were observed between the severity of CT scan and raised inflammatory markers among COVID-19 patients in a study conducted by Saeed et al. [1]. In that study, elevated CRP levels and D-dimer levels were found to correlate with the CTSS (p<0.0001), which is statistically significant. This suggests that these parameters could be used as a prognostic indicator, where higher levels are seen in more critical conditions. A regression analysis by Wang et al. showed that CRP was significantly associated with the aggravation of nonsevere COVID-19 patients, with an area under the curve of 0.844 (95% confidence interval) [11]. Consistent with this study, our results indicated that there were statistically significant correlations between CTSS, CRP, and D-dimer levels among COVID-19 patients. However, it is not yet clear whether this increase is related to the direct effect of the virus or the systemic inflammatory response [27].

Ferritin is a key mediator of immune dysregulation, especially under extreme hyperferritinemia, via direct immunosuppressive and pro-inflammatory effects, contributing to the cytokine storm. In one study with 20 COVID-19 patients, it was found that individuals with severe and very severe COVID-19 exhibited increased serum ferritin levels [28]. Another study revealed that in patients who died from COVID-19, ferritin levels were high upon hospital admission and throughout their hospital stay. Similarly, serum ferritin was noted to be an immune mediator, with its level indicating the severity of the disease [29]. By contrast, our study did not show any correlation between CTSS and serum ferritin levels in COVID-19 patients (p=0.260).

D-dimers are one of the fragments produced when plasmin cleaves fibrin to break down clots. The assays are routinely used as part of a diagnostic algorithm to exclude the diagnosis of thrombosis. However, any pathologic or non-pathologic process that increases fibrin production or breakdown also increases plasma D-dimer levels [30]. Examples include deep vein thrombosis/pulmonary embolism, arterial thrombosis, disseminated intravascular coagulation, and conditions such as pregnancy, inflammation, cancer, chronic liver diseases, post-traumatic and surgery status, and vasculitis [31]. Presumably in SARS-COV-2, the observed D-dimer elevation signifies a hyperfibrinolysis state and increased inflammatory burden induced in SARS-COV-2 infection. Yao et al. reported that D-dimer levels significantly increased with the increasing severity of COVID-19 as determined by clinical staging (Kendall's tau-b=0.374, p=0.000) and chest CT staging (Kendall's tau-b=0.378, p=0.000). High median D-dimer levels correlated with poor outcome (mortality). A D-dimer level of >2.14 mg/L predicted in-hospital mortality with a sensitivity of 88.2% and a specificity of 71.3%. Consistent with this, we also observed a significant correlation between D-dimer and CTSS (p=0.020) [32]. Several studies have proposed LDH as a parameter that could be correlated to disease severity. In our study, we found a statistically significant correlation between LDH and CTSS (p<0.001), which was also reported by Leonardi et al. [33]. The high LDH level in COVID-19 in severe cases suggested that LDH may be associated with lung injury and tissue damage, warranting an investigation to identify the potential mechanism.

Similar correlations were demonstrated in previous studies to have a significant association with age more than 65 years, hypertension, LDH, and D-dimer with severe COVID-19 disease [34-37]. In the same study, the levels of LDH, NT-pro B-type natriuretic peptide, and D-dimer and serum cytokine levels of IL-6 were significantly higher in severe patients than in nonsevere COVID-19 patients (p<0.01). The study also revealed that hyperglycaemia was related to increased mortality in patients with COVID-19 [38].

Clinical outcomes

Although the in-hospital stay for the majority of subjects was between three and four weeks, the duration of CCU stay among most patients in our study was one week. Length of hospital stay and CCU stay were found to have significant correlations with CTSS in our study, suggesting that an increased score increased the duration of the hospital stay in these patients. Saeed et al. [1] reported similar observations. An increased death rate was reported among those with more severe scans. Chung et al. and Huang et al. reported that higher CTSS were found in patients admitted to CCU [39,40]. Das et al. concluded that higher chest CT scores correlated with poor prognosis and high mortality in patients with Middle East respiratory syndrome (MERS) [41]. However, in our study, patients with a CTSS of less than 8 were also admitted to the CCU. CTSS had little effect on discharge and death rates even though it was statistically significant (p<0.05).

## Conclusions

Patients with COVID-19 present to CCU with varying degrees of severity of illness. The results of our study suggest that there is a statistically significant correlation between CTSS and laboratory parameters such as CRP, D-dimer, NLR, LDH, and IL-6. Although it is not the gold standard for diagnosis, CTSS could be of great help in the screening and early identification of the disease, especially in those patients awaiting an RT-PCR report or with a negative RT-PCR, which will lead to appropriate isolation and treatment measures. It could also help in assessing the progression of the disease, alter the course of management at the earliest possible time, and provide better prognostication for COVID-19 patients. Repeat CTSS during the course of disease treatment could indicate the progression of the disease as it correlates with several other parameters. Finally, a larger multi-center study with better validity is necessary to determine the validity of CTSS in diagnosing disease in COVID-19 patients and its association with various laboratory and clinical parameters and outcomes.
